# The Language of “Rate of Change” in Mathematics

**DOI:** 10.3390/ejihpe11040113

**Published:** 2021-12-06

**Authors:** Evgenios Avgerinos, Dimitra Remoundou

**Affiliations:** Mathematics, Mathematical Education and Multimedia Laboratory, Department of Education, University of the Aegean, 85100 Rhodes, Greece; remoundou@aegean.gr

**Keywords:** calculus, rate of change, language of mathematics, mathematics education

## Abstract

Language is an essential aspect of teaching and learning mathematics. It is necessary for communication, the transmission of concepts and ideas, and the formation of meaning of mathematical concepts. In mathematics, besides symbols, which are usually common among different languages, words and expressions are used, which may invoke different concept images to students in various languages. Some words are used in mathematics and in everyday language with different meanings, while others are used only in mathematics or in mathematics and other disciplines in similar but non-identical ways. In Mathematical Analysis, the used vocabulary is gradually enhanced, and the concepts are defined in a more formal way. In the current study, the language used regarding mathematics of change is examined, focusing on “rate of change”, and its relation to misconceptions among students.

## 1. Introduction

Learning the language of mathematics is part of learning mathematics [1,2]. The language of mathematics may be considered a precise, global language with no ambiguities. Mathematical terms are defined rigorously, and the symbols are mostly common among different languages. 

Nevertheless, the way language is used in mathematics differs from the everyday language and may cause challenges to learners [2]. Ambiguities regarding technical vocabulary as well as other linguistic challenges, including grammar, may confuse students and impact their understanding of mathematics.

In a social activity such as education, language is not always strictly used. Sometimes, words are omitted, phrases are shortened, and gestures and pronouns are used instead of the mathematical terms. Teachers try to help students understand new mathematical concepts and move from informal, everyday language to formal language of mathematics [1,2].

Use of known words in a stricter way in mathematics or ambiguities should not be always considered a problem. In the social discourse of a classroom, formal and informal uses of language are combined, and ambiguity provides opportunities for teachers and students to extend their thinking and understanding of the language of mathematics [3].

Rate of change is an important concept of mathematics, used in everyday life and applied in a variety of disciplines. It is studied in school curriculum and in more advanced university courses. The concepts related to rate of change are expressed with different terms and expressions, some of which are not clearly defined in school textbooks, while others are partially defined and enhanced later.

Research on students’ understanding of rate of change has revealed difficulties in the conceptualization of rate and its interpretation in physical phenomena [4,5,6,7]. Some studies provide indications that some difficulties are related to the used language [5,8]. 

The main objective of the current study is to examine the language used in mathematics, particularly in tasks involving the concept of “rate of change”, and to locate possible misconceptions related to the use of language. As language is important in teaching and infers the knowledge and the meaning of concepts that students construct, the vocabulary used for “rate of change” and related concepts is examined. Change and “rate of change” are studied as fundamental topics of Mathematical Analysis and the linguistic challenge associated with them is addressed. Specifically, misconceptions of rate, slope, tangent, derivative, and velocity are examined regarding the language used to express them in school mathematics. The vocabulary used regarding rate of change is very rich with complicated expressions, and some terms are not clearly defined in school textbooks. A new term as an alternative to “rate of change” is proposed for use in mathematics, sciences, and economics, which should be clearly defined and used throughout the disciplines.

## 2. The Role of Language in Mathematics Education 

Mathematics is considered as a global language, which students must master to be able to understand the concepts and communicate their ideas fluently. Mathematics language consists of words, numbers, symbols, and diagrams and is the key to accessing mathematical concepts [1]. In mathematics education, it involves the ability to use words to explain concepts, justify procedures, and communicate mathematically [1]. 

The term concept image as expressed by Tall and Vinner [9] is used to describe “the total cognitive structure that is associated with the concept, which includes all the mental pictures, associated properties and processes”. Concept image may be a visual representation of a concept or a collection of impressions or experiences [10]. Students construct a concept image of a mathematical concept according to their personal experiences, which may diverge from the mathematical concept as mathematicians or teachers understand it [11]. The term concept definition is used as a form of words used by the learner to define the concept [9].

Students have spontaneous conceptions of mathematical concepts before the formal teaching [12]. Part of their conceptions is the meaning of mathematical terms according to their experiences and the use of everyday language [12]. Informal language and terms of everyday language known to students are often used by educators to explain new mathematical concepts. 

The meaning of terms and expressions that students already know may differ from the formal mathematical language and result in misconceptions. Moreover, the spontaneous conceptions remain even at an advanced level of learning [12] and are difficult to change, especially when the mathematical terms are used in everyday language and their mathematical definition is not clearly stated [10]. 

Sometimes, students do not have the same concept image for a mathematical concept as the mathematicians, even if they use the same language to describe it [13]. The definition of a mathematical concept is not always understood by students the way teachers believe, and the concept definition students form may differ from the formal definition [9]. There are indications that students use their concept image instead of the concept definition to solve tasks [10]. Moreover, students tend to use informal language to express themselves and may use mathematical terms in contexts where they have a colloquial meaning [14].

For students to form a concept image that is similar to the image mathematicians and teachers have, students and teachers should share a common code of communication. To help students learn and use the language of mathematics, educators should be aware of the difficulties related to terminology [1]. The formation of a productive concept image is not achieved by just stating the concept definition—it should be related to everyday life with examples and non-examples [10].

Lexical ambiguity is the result of assigning different meanings in a word or phrase and may appear when a word of everyday language is used as a technical term in a discipline [3]. Rubenstein and Thompson [15] have reported 11 categories of difficulties related to the learning of mathematical language: (a) words common in mathematics and everyday language with different meanings in the two contexts, (b) words common in mathematics and everyday language with comparable meanings, but the mathematical meaning is more precise, (c) terms used only in mathematics, (d) words used in mathematics with more than one meaning, (e) terms used in mathematics and other disciplines with different technical meanings, (f) mathematical word homonyms with everyday language words, (g) words that are related, but students confuse their distinct meanings, (h) English words that are translated in different ways in another language, and vice versa, (i) English spelling and usage irregularities, (j) mathematical concepts that are verbalized in more than one way, and (k) use of an informal term as if it was a mathematical term.

Linguistic challenges and lexical ambiguities are apparent in many fields of mathematics. The completely or slightly different meaning of words in mathematics and everyday language or other disciplines has been subject of research and reflection in mathematics education [16]. There is some recent research on lexical ambiguity regarding mathematical terms in statistics [17,18] and algebra [19,20]. 

Regarding Mathematical Analysis, there are references in research to students’ misconceptions related to language [10,12]. There are indications that more emphasis in Calculus teaching is placed on procedures instead of the concepts [21]. Students can solve problems of Calculus algorithmically by memorizing standard procedures, but they have not acquired the meaning of the concepts [11]. Some of the misconceptions in concepts of Calculus seem to be related to the language used, e.g., regarding the terms limit or tangent [10,12]. Moreover, in Calculus, long phrases are often used to correctly express a concept, and a shortening of a phrase may result in completely different meaning.

Language skills are essential in word problems solving, firstly to understand the problem and then to express the solution [22]. There are indications that students’ language proficiency is related to their proficiency in solving mathematics word problems [23]. Calculus is widely used to model and solve problems of sciences. Language is reported by students as one of their main difficulties in solving application problems [24].

The role of school textbooks is vital in education. Teachers use them to decide what to teach and they are students’ main study tool. The language used in textbooks affects students’ understanding and may result in misconceptions [25]. Research on the language used in mathematics and the possible misconceptions it may cause would help improve school textbooks and teaching.

Ambiguous meanings are not necessary obstacles in education and particularly mathematics. If teachers are aware of possible meanings that are recalled by new terminology in mathematics, they could take advantage of the language used by students and connect it to mathematical concepts [26].

## 3. Method

In the present study, the language used in mathematics regarding concepts of Mathematical Analysis as “change” and “rate of change” is addressed. The study was triggered by the difficulties observed among students when describing “rate of change” and the belief of mathematics educators that students do not understand the language used in “rate of change” tasks [27]. The study focuses on the language used in mathematics, in tasks involving “rate of change” and other concepts related to it. Aspects of “rate of change” are discussed and presented in a concept map to describe the main concepts of the study and emphasize the variety of concepts and terms. In addition to “rate of change”, concepts related to it such as ratio, slope, tangent, derivative, and applications of “rate of change” in contexts are addressed regarding linguistic challenges.

Literature on research on mathematics education has been reviewed regarding the language used in “rate of change” tasks, and possible misconceptions it may cause to students. A non-systematic literature review method was used. The publications used in the current study were selected among an extensive literature collection on mathematics education, the role of language, and concepts related to “rate of change”.

The findings of the selected pieces of research have been analyzed, compared, and synthesized. Although the main focus of many of the reviewed studies was not the use of language, the interpretation from this perspective gives prominence to the role of language in mathematics education and related difficulties. Moreover, references to “rate of change” in mathematics textbooks and education material were considered.

Searching known scientific databases led to some results, but they were less than satisfactory. The difficulties of a systematic literature review regarding the research questions were that “rate of change” as a key search term results in many publications not related to mathematics education, while most of the publications found by the term “language”, even when combined with “mathematics education”, refer to second language or English as a foreign language.

Moreover, although there are only a few publications that address “rate of change” and language in mathematics education, the use of language has been revealed as a side result in a few studies. Consequently, many publications that are related to the research questions of the current study do not appear in search results.

## 4. Results

### 4.1. Rate of Change Aspects

Mathematical Analysis expresses the mathematics of change. Mathematical Analysis, as a name of an area of mathematics, is not clear as Geometry or Algebra, and may be used in different contexts with different meanings. 

A part of the vocabulary used in Mathematical Analysis is not new to students. There are words already known and used in everyday language. For example, “limit” and the expression “tends towards” have many different meanings for students [12]. These ambiguities result in a concept image that may conflict with the formal definition of the concept [12]. 

Continuity suffers from lexical ambiguity, as well. Expressions from everyday life such as “it rained continuously all day” or “the railway line is continuously welded” are used by teachers as an introduction to continuity [9]. However, such expressions may result in the idea that the graph of a continuous function has “no gaps” [9].

Rate of change as a fundamental concept of Mathematical Analysis is rich in meanings and complex for most students. To describe a dynamically changing phenomenon, besides the study of the change of one quantity, the way this change happens is also required.

Rate of change is taught in different levels of education, from school to university and in various disciplines. Besides mathematics, it is used in other sciences and has applications in physics, economics, biology, mechanics, and electronics. Some well-known and frequently used rates of change are velocity, acceleration, and power; rate of change of kinetic energy, of dynamic energy, and of momentum; as well as economical concepts such as marginal cost and profit. 

The language used to describe dynamically changing phenomena indicates the understanding and the image one has for the concepts. Students may form different images of rate of change, according to their age, grade, and previous experiences. Some misconceptions and errors may result from the language used in school textbooks and by educators [6]. 

**Figure 1:**
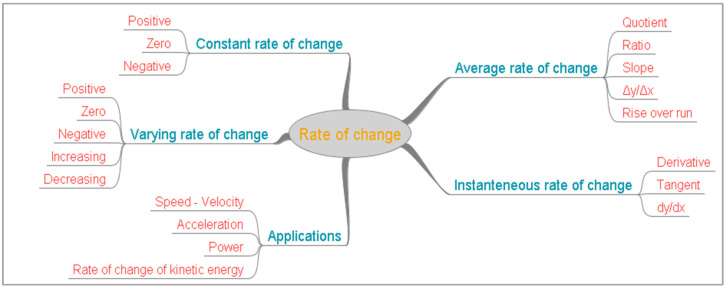
A concept map of rate of change.

Figure 1 is a concept map of rate of change and presents some of the concepts related to rate. For some of the concepts, more than one word is needed to precisely express their meaning and if they are omitted, the concepts are not clear. 

The English word “rate” has the meaning of value or ratio. In Greek, the term is used in music, art, architecture, mathematics, physical sciences, economics, biology, and everyday life. It usually has the meaning of a periodical motion. 

Ratio, quotient, accumulation, and proportionalities are basic concepts for understanding rate of change [28]. Thompson [29] refers to the terms ratio and rate, noticing that the use of two different terms implies that there are two different concepts, but in practice, they are used interchangeably. According to the researcher, the two terms are used in education and research without definition and without clear distinction [29].

In mathematics, rate is used in Statistics, Mathematical Analysis, and Algebra. Students are asked to express and explain rates in mathematics areas with precise language and distinguish it from the terms used in everyday language.

Rate of change can be average or instantaneous. Average rate of change is related to the ratio of differences or the slope, while instantaneous rate of change is the derivative. It may be constant, in which case, the instantaneous and the average rate of change are the same or varying. The value of rate of change may be positive, negative, or zero, and moreover, it may be increasing or decreasing. Moreover, the rate of change of a function has a rate of change itself. All these aspects, with the used words and adverbs, form a complicated image of rate of change. 

One of the linguistic challenges of mathematics are the dense noun phrases that participate in relational processes [2]. Speaking of rate of change includes such phrases that are difficult to interpret. When students describe dynamically changing phenomena, they have to combine nouns, such as “derivative”, “rate of change”, “slope”, with adverbs, such as “at a point”, “on an interval”; verbs, such as “increase” and “decrease”; or adjectives, such as “positive/negative” and “general/specific” [30]. 

The rate of change is usually related to specific contexts, where the double or triple use of the proposition “of” presents another challenging factor. A not unusual expression of a rate of change problem may be “The rate of change of volume of water in the tank is increasing at a decreasing rate”, which requires familiarity of the student with the language to be able to interpret. In some tasks, shortened expressions such as “rate of increase/decrease” are used.

For the description of the behavior of a function, phrases such as “increasing at a decreasing (or an increasing) rate” or “decreasing at an increasing (or a decreasing) rate” are used, which are quite confusing [31]. The contrasting adjectives are not easy to interpret. The difficulties many students face with such expressions is apparent in studies, in which students fail to distinguish the behavior of the function and the behavior of its derivative [32]. This is mostly observed in studies with tasks of rate of change in a specific context, in which students express the behavior of the function in that context, but have difficulties using more formal mathematical language and confuse the behavior of the function with that of its rate of change [8].

Some researchers have proposed to split these long phrases in two, one describing the behavior of the function and one for the derivative, such as “The function is increasing (decreasing) and its rate of change is increasing (decreasing)” [32].

Zandieh and Knapp [33] examined the role of metonymy in student reasoning about derivative, pointing out that students may believe that a shortened phrase is accurate and true or that both the extended and shortened phrase are true. Metonymy is often used in Calculus, and it is accepted in education, but the excessive use of shortened expressions may hide information that is not trivial and may be related to a lack of understanding [33].

Some rates of change, such as velocity, acceleration, power, and electric current in physics, express another physical magnitude. In these cases, a new term is used instead of rate of change. Even if it seems easier for students to understand these concepts, the different terms that are used may cause misunderstandings and it is more difficult to relate the concepts to rate of change and its properties. It is simpler to speak about acceleration instead of rate of change of velocity, but it is harder to conceptualize the connection between the two magnitudes. Students seem to conceptualize the concepts as new entities and not as the measure of two covarying quantities [34].

A common misconception described in literature regarding rate of change is to express the magnitude but not the sign of rate [8]. Negative rates of change that increase are difficult to express, because students confuse the absolute value which decreases with the signed value which increases. This is more intense when describing phenomena for which the everyday language refers to the magnitude of the quantity [8,32].

The term “constant” for describing rate of change and function may cause misconceptions. A rate of change that is “increasing constantly” may be conceptualized as constantly increasing (increasing all the time), while in mathematical terms, this means that it is increasing at a constant rate [8].

Another mathematical term that may cause misconceptions due to lexical ambiguity is “average” [13]. Students’ experiences with the term “average” in everyday language conflict with its use in Statistics and Calculus [13]. Average may refer to arithmetic mean, median, or mode in statistics or may express something normal or usual in everyday language [13]. In Bezuidenhout’s [11] research, some university students added up a number of g(x) or g’(x) values and divided by the number of instances to find the average rate of change. Average in Calculus is confused with the arithmetic mean in statistics. Average rate of change, average value of a function, and arithmetic mean are confused and cause misconceptions for many students [11], along with average rate of change and instantaneous rate of change [5]. Students’ concept images of average prevent students from thinking about average rate of change as a quantity changing at a constant rate with respect to another quantity [13].

### 4.2. Concepts Related to “Rate of Change”

#### 4.2.1. Slope

Average rate of change of a function between two points is the slope of the secant line in these points, while instantaneous rate of change in a point is the slope of the tangent line at this point. In linear functions, rate of change equals the slope of the line. Understanding of slope of linear functions is crucial for the conceptualization of derivative and rate of change [35]. 

The terms and symbolism used for slope in school textbooks are ambiguous and may cause misconceptions [36,37]. The term “slope” is associated with the words “steep”, “elevation”, “descent”, and “inclined” from everyday language [38]. Students hold an image of slope, as a roof, a mountain, or another inclined surface [39]. Besides mathematics, slope is used in other scientific disciplines, such as art, architecture, mechanics, and physical sciences [38]. Students have an intuitive understanding of slope from their experiences, which needs to be transformed to the formal definition in mathematics.

In mathematics, slope can be conceptualized geometrically, algebraically, trigonometrically, and in Calculus [40]. The phrase “rise over run” is used as a mnemonic rule to remember how to compute slope and students who use it correctly seem to understand slope as a ratio or rate [39].

Slope, rate of change, and steepness are sometimes used as having the same meaning in textbooks [6]. Coe [41], in a study on mathematics teachers, noted that even experienced teachers have difficulties expressing the connection between division, rate, and slope.

#### 4.2.2. Tangent

Students encounter the notion of tangent in different contexts in mathematics, as a tangent to circle in Geometry, tangent line of conic sections in Analytic Geometry, and tangent line to a graph in Calculus [42]. While the used term remains the same in the different contexts, the definition changes. 

The word “tangent” stems from the Latin adjective “tangents”, which means touching [43]. Students learn first about tangent lines in the context of geometry, as a tangent to a circle. Teachers and some textbooks, attempting to clarify the concept of tangent line, use everyday language and experience of the students and define tangent as the line that touches the circle at exactly one point [25].

In Calculus, the concept of tangent is extended and includes cases in which the tangent line may cross the curve at another point [43]. Moreover, the term tangent is used in trigonometry, as tangent of an angle. The same term is used because the tangent of the angle in a unit circle equals the segment of the tangent line to the circle [43]. Students may confuse the idea of tangent line with the function y = tan(x), and tangent of an angle [44].

Students tend to use the “touching” cognitive model of a tangent in Calculus [25]. A common misconception of students is that a tangent line to a more general curve may only meet the curve at one point and may not cross the curve at that point [10,25,42].

#### 4.2.3. Derivative

Tangent, slope, and derivative are confusing for students, and it has been reported that some students talk about derivative as the tangent line instead of its slope [30,33]. There are indications that students have a better understanding of the derivative if their concept image of tangent includes the limiting position of secant lines, instead of the touching cognitive model [45]. In one study, mechanical engineering students were found to think of derivative in terms of rate of change and mathematics students in terms of tangents [46].

Shortening of long, complicated phrases may result in misconceptions. In Amit and Vinner’s [21] study, the researchers noticed that the definition of the derivative at a point as “The derivative of a function at a certain point is the slope of the tangent to the graph of the function at this point” may result in “The derivative is the tangent to the function at a certain point”. Omitting slope in the definition results in different meaning and it is not clear if students understand the way tangent is used or understand derivative as the equation of the tangent.

The word “derivative” refers both to derivative at a point and derivative of a function [30]. Category (g) of Rubenstein and Thompson’s [15] work refers to words that are related, but students confuse their distinct meanings. Moreover, the challenge is more intense when speaking about phrases that are similar but refer to different concepts, such as “derivative of a function at a point” and “derivative function” or “average rate of change of a function” and “average rate of change function”. 

#### 4.2.4. Speed-Velocity

Speed is the rate of change of distance travelled over time. Students sometimes use the term speed to describe rate of change, while an analogy of rate of change to speed in a problem may help students interpret the problem [33]. As with constant and average rate of change, misconceptions about constant and average speed have been reported [5]. 

A confusion has been observed in literature when the same word is used for magnitude and the signed value of a quantity [32]. In such cases, attention should be paid to the language used to help students understand and communicate about phenomena with negative rates [32].

In English, there are two different words: speed, which describes the magnitude, and velocity, which expresses the signed value. Contrary, in the Greek language, the same word is used for speed and velocity in everyday language, and in textbooks, it is not always clear which concept is being referred to. 

Other concepts that are not clearly distinguished for many students are the quantity and the change in a quantity and change and rate of change [47]. In these cases, the same words are used in phrases that express different concepts. Moreover, in the Greek language, the word “analogia” is used in mathematics and other fields, such as biology and linguistics, and in everyday language. In mathematics, it expresses proportionality, while it also means analogy, as described by Polya [48].

## 5. Discussion

Rate of change is a general concept that involves many aspects and can be conceptualized in different ways and in various contexts. In Greece, as in other countries [28], rate of change and covariation are not emphasized in school textbooks and curricula of primary and secondary education [37], and they seem to cause significant difficulties among students. 

The literature review on the effects of language on the construction of concepts of Mathematical Analysis and especially rate of change implies that there are some difficulties related to language. The findings of the current study are in accordance with findings reported for other concepts of mathematics [18,19,20]. Terminology regarding rate of change has long sentences, ambiguities, terms used in everyday language, and terms used without definition or defined in different ways. 

As pointed out in research on mathematics education [2], it appears that the way the language is used in mathematics regarding the concept of rate of change differs more or less significantly from the colloquial language. Most of the terms discussed and used when speaking about rate are words common in mathematics and everyday language with comparable meanings, e.g., rate, slope, constant, and average. This category of ambiguity may cause significant misconceptions among students as they already hold a concept image for them [2]. 

Lack of familiarity with the vocabulary used in Mathematical Analysis results in errors and poor understanding of the concepts. Words used in colloquial language with different meanings need to be clarified. Nevertheless, students’ experiences and words from everyday life may be carefully used to trigger the introduction of new concepts.

The study reinforces the importance of the role of educators in clarification and understanding of mathematical concepts [17]. If teachers are aware of the potential problems caused by the used language, they can find ways to overcome the difficulties and clarify statements that lead to misconceptions [31]. Emphasis should be placed on the correct use of language in the definition of terms, properties of concepts, and problem solving. Moreover, students should have opportunities to express orally and in writing their conceptions and participate in classroom discourse to enhance their mathematical linguistic abilities, and, as a result, their understanding of concepts. 

Modeling dynamically changing events requires familiarity with the language of change and rate of change. Activities related to real-world problems, modelling, and analysis of phenomena could help students enhance their linguistic abilities and mathematical terminology, and, as stated in other studies [8], their reasoning about changing phenomena. Students should actively participate in class discourse to familiarize themselves with the language of mathematics. Interpretation of phenomena in physics or other sciences may help students better understand phenomena and the mathematics behind them. Concept maps, examples, and non-examples can be used as tools to reveal similarities and differences between terms, expressions, and notations in mathematics. 

Following the current study, we adopt the view of researchers that covariation should be included in early education curricula [4,28]. Concepts related to rate of change, such as slope, variation of quantities, constant, and average rate of change, could be taught in early secondary education as part of mathematics of change, extended later to derivative and instantaneous rate of change. A robust understanding of a concept image of rate could be generalized to derivative and more advanced topics of Calculus. 

Instead of “rate of change”, another term could be used in mathematics, sciences, and economics. Using one word would simplify some complicated expressions. Moreover, the phenomenon of omitting words to make the sentence smaller would be avoided, as well as the double use of the preposition “of”. One proposal for a one-word term that would express “rate of change” is the term “variationality”. If a specific term is clearly defined and used throughout the disciplines, students could form a general, robust image of “rate of change”, integrating the various aspects and emphasizing its covariational nature. As a result, learners could conceptualize quantities of sciences as different aspects of the same concept and better understand their properties.

The findings of this study must be seen considering some limitations. The literature review was not systematic, and the sample of the publications was selected by the authors based on broader research on the concept of “rate of change” in mathematics education. As a result, there may be studies related to the research questions and not taken into account. The current study could be extended and combined with a more systematic literature review.

## 6. Conclusions

The present synthesis of selected publications in relation to the references on mathematics textbooks has revealed some linguistic challenges regarding the use of “rate of change” in mathematics education. Lexical ambiguities, long and complex phrases, etc., may impede the understanding of the concepts or cause misconceptions. Mathematics textbooks writers and educators may benefit from the mentioned complexities and find ways to enhance students’ understanding through written mathematical texts and discourse in classrooms.
